# Regulatory and Activated T Cells in Human *Schistosoma haematobium* Infections

**DOI:** 10.1371/journal.pone.0016860

**Published:** 2011-02-10

**Authors:** Norman Nausch, Nicholas Midzi, Takafira Mduluza, Rick M. Maizels, Francisca Mutapi

**Affiliations:** 1 Ashworth Laboratories, School of Biological Sciences, Institute for Immunology and Infection Research, University of Edinburgh, Edinburgh, United Kingdom; 2 National Institute of Health Research, Harare, Zimbabwe; 3 Department of Biochemistry, University of Zimbabwe, Harare, Zimbabwe; The George Washington University Medical Center, United States of America

## Abstract

Acquired immunity against helminths is characterised by a complex interplay between the effector Th1 and Th2 immune responses and it slowly manifests with age as a result of cumulative exposure to parasite antigens. Data from experimental models suggest that immunity is also influenced by regulatory T cells (Treg), but as yet studies on Treg in human schistosome infections are limited.

This study investigated the relationship between schistosome infection intensity and the two cell populations regulatory T cells (Treg: CD4^+(dim)^CD25^+(high)^FOXP3^+^CD127^low^), and activated (Tact: CD4^+^CD25^+^FOXP3^−^) T cells in Zimbabweans exposed to *Schistosoma haematobium* parasites. Participants were partitioned into two age groups, young children (8–13 years) in whom schistosome infection levels were rising to peak and older people (14+ years) with declining infection levels. The relationship between Tact proportions and schistosome infection intensity remained unchanged with age. However Treg proportions rose significantly with increasing infection in the younger age group. In contrast Treg were negatively correlated to infection intensity in the older age group.

The relative proportions of regulatory T cells differ significantly between young individuals in whom high infection is associated with an enhanced regulatory phenotype and older infected patients in whom the regulatory response is attenuated. This may influence or reflect different stages of the development of protective schistosome acquired immunity and immunopathogenesis.

## Introduction

Schistosomiasis is a major human parasitic disease caused by different species of trematode parasites of the genus *Schistosoma*. Urogenital schistosomiasis caused by *S. haematobium* is the most prevalent form in sub-Saharan Africa where it is associated with pathology ranging from hematuria to more severe conditions such as hydronephrosis and bladder cancer [1].

The role of acquired immunity in reducing human schistosome infection intensity and pathology has been subject to intense study [2], [3], [4]. Early studies suggested that anti-schistosome immune responses fell into a clear Th1/Th2 dichotomy with resistance to infection being associated with Th2 responses [5], [6], but mounting evidence shows this dichotomy cannot adequately explain the balance between susceptibility and resistance to infection [7], [8]. For example, we have previously shown that neither Th1 nor Th2 cytokine responses in *S. haematobium-*infected Zimbabwean children show a clear pattern with infection intensity [9], [10] supporting similar reports on *S. manosni* vaccine candidates [11] and other immuno-epidemiology studies (reviewed in [12], [13]).

The discovery that a regulatory subset of CD4^+^ T cells (Treg) modulates the activity of effector Th1 and Th2 responses in mouse models of schistosomiasis [14] has led to suggestions that it is the balance between effector and regulatory responses including Treg, which determines the outcome of murine helminth infections [15], has also been postulated for human helminth infections [16], [17]. This concept is not unique to helminth infections; regulatory T cells have been invoked across a wide range of infectious diseases including fungal, bacterial and viral infections [18], [19], [20], [21], [22] and non infectious diseases such as allergies or cancer [23], [24], and modulation of immunopathology by regulatory T cells has been reported in human and mouse models [25], [26], [27], [28].

There have been several studies of the phenotype and role of Treg cells in mouse models of helminth infections [15], [29], [30], [31] including schistosomiasis [27], [32], [33], [34], but to date there is a paucity of studies of human Treg responses in helminth infections. Human Treg were initially identified as those expressing the highest levels of CD25 [35], and using this marker it was reported that regulatory and effector cells show an inverse relationship in schistosomiasis [36]. In addition a study in Kenyan children found differences in activated T cells and memory regulatory T cells between individuals with *S. mansoni* only compared to individuals concurrently infected with *S. mansoni* and *Plasmodium falciparum* [37].

The discovery of the forkhead/winged helix transcription factor FOXP3 as a marker for these cells allows a more accurate characterization of Treg [38], [39], [40]. Some studies suggested that human FOXP3^+^ cells (in contrast to mouse FoxP3^+^ Treg) are functionally heterogeneous [41] and that transient expression of FOXP3 on activated T cells does not correlate with suppressive function [42]. However FOXP3 is still considered the most accurate marker for naturally occurring Treg [43]. Recent studies have further characterized the phenotype of Treg by the expression of low levels of CD127 [44] and slightly diminished levels of CD4 [45]. By using a combination of these markers we have investigated the relationship between Treg, effector T cells and schistosome infection intensity. The aim of the study was to determine if the percentage of activated T cells (Tact, defined as CD4^+^CD25^+^FOXP3^−^) or regulatory T cells (Treg, defined as CD4^+^CD25^+(high)^FOXP3^+^CD127^dim^) differed with schistosome infection intensity.

## Materials and Methods

### Ethics Statement

Permission to conduct the study in the region was obtained from the Provincial Medical Director and institutional and ethical approval was received from the University of Zimbabwe's Institute Review Board (UZIRB) and the Medical Research Council of Zimbabwe (MRCZ) respectively. Only compliant participants were recruited into the study and they were free to drop out at any point during the study. At the beginning of the study, participants and their parents/guardians had the aims and procedures of the project explained fully in the local language, Shona, and oral consent was obtained from participants and parents/guardian before parasitology and blood samples were obtained. Oral informed consent was obtained because of the high levels of illiteracy and cultural reasons [46]. Both the UZIRB and MRZC approved the use of oral consent. Upon oral consent participants were enrolled in the study with a written record of their name, age, sex and case number, this served as both the record of oral consent and enrolment record.

### Study population and parasitology

The study was conducted in the Mashonaland East Province of Zimbabwe (31^o^30′E; 17^o^45′S) where *S. haematobium* is endemic and where the participants have been participating in an ongoing study of the immuno-epidemiology of human schistosomiasis [47], [48]. The villages were selected because health surveys regularly conducted in the region showed little or no infection with other helminths and a low *S. mansoni* prevalence (<5%) [49], [50]. The two villages selected (Goromonzi and Mutoko) had not been included in the National Schistosome Control Programme and therefore participants had not received antihelminthic treatment for schistosomiasis or other helminth infections meaning that their natural immune responses could be studied in the absence of drug-altered schistosome responses [51]. The main activity in these villages is subsistence farming and human water contact is frequent with at least 4 contacts/person/week (assessed by questionnaire, adapted from [52], [53]) due to insufficient safe water and sanitation facilities. Drinking water is collected from open wells while bathing and washing is conducted in two main rivers in the villages. The schools surveyed in the two villages were all in close proximity to rivers.

Helminth infection intensity was assessed by microscopic examination of urine and stool samples. Urine specimens were processed by urine filtration following a standard method originally described by Mott *et al*. [54] for diagnosis of *S. haematobium*. Fresh stool specimens were processed by the Kato-Katz technique and subsequently analysed by microscopy for intestinal helminths including *S. mansoni*, hookworm, *Ascaris lumbricoides* and *Trichuris trichiura*. The formol-ether concentration method was performed on 50% of the samples to confirm results obtained by the Kato-Katz technique [55]. Stool and urine specimens were collected on three consecutive days.

In order to be included in the cohort, participants had to meet all the following criteria which are comparable to other studies performed for human Schistosomiasis [36], [56], [57]: 1) be life-long residents of the schistosome-endemic area; 2) not have received prior treatment for helminth infections; 3) have provided at least 2 urine and 2 stool samples on consecutive days; 4) be negative for intestinal helminths including *S. mansoni* and negative for *Plasmodium* parasites as determined by blood smears; 5) have given a blood sample for the collection of peripheral blood mononuclear cells (PBMC). Forty-nine participants aged 8–60 years met these criteria and formed our study population. Questionnaire studies indicated that there were no significant differences in water contact measures (frequency or duration) between the age groups. After collection of all samples, all participants were offered treatment with the recommended dose of praziquantel (40 mg/kg of body weight).

### Collection of PBMC

Twenty millilitres of venous blood was collected in heparinised tubes of which 5 ml was used for serological assays as well as microscopic detection of malaria parasites. Fifteen ml were used to collect peripheral blood mononuclear cells (PBMC) through density gradient centrifugation using Lymphoprep™ (Axis-Shield, Cambridgeshire, UK). These PBMC were subsequently, enumerated, cryo-preserved and stored in liquid nitrogen in Zimbabwe prior to freighting to Edinburgh in dry shippers for assay.

### Phenotyping of PBMC

Thawing of cryopreserved PBMC was performed by rotating cryovials in a 37°C water bath until a small crystal was remaining in the cell suspension. Medium (RPMI 1640 supplemented with 10% Fetal bovine serum, 2 mM L-glutamine and 100 U Penicillin/Streptomycin; all Lonza, Verviers, Belgium) was slowly added to the PBMC. Cells were washed twice with the corresponding medium, counted and viability assessed using trypan blue (Sigma-Aldrich, Dorsert, UK). Afterwards cells were washed with Dulbecco's-PBS (Lonza) and surface stained with the following antibodies: Alexa488-conjugated anti-CD4 (clone OKT-4), PE-Cy7-conjugated anti-CD127 (clone eBioRDR5; all from eBiosciences, San Diego, USA), PE-conjugated anti-CD25 (clone M-A251, BD Biosciences, San Jose, USA). Intracellular staining was performed using APC-conjugated anti-FOXP3 (clone PCH101, eBioscience) and the intracellular staining set from eBioscience following the maufacturer's instructions. Stained cells were analysed on a FACSCalibur™ using CellQuestPro™ software (BD Biosciences).

### Statistics

In an initial analysis was determined if the proportion of Treg and Tact relative to the total CD4^+^ T cells varied with host age. This analysis was conducted using a multivariate analysis of variance (MANOVA) allowing for sex (categorical; male, female), and residential village (categorical; Goromonzi, Mutoko). T cell percentage data were arcsine square root transformed to allow the use of parametric tests [58].

Subsequently the relationship between the proportions of Treg and Tact relative to total CD4^+^ T cells and infection intensity was determined using an analysis of variance (ANOVA). In this statistical test, the dependent variable was infection intensity (log _10_(x+1) transformed), and the independent variables were Tact and Treg transformed as above. The possible confounding variables sex, village (categorised as before) and age (categorical; age group 1 (8–13 years), and age group 2 (14+ years)) were allowed for by using sequential sums of squares. Following the ANOVA the relationship between the between the two T cell subsets and the infection intensity was analysed using residuals for infection intensity (log_10_(x+1) transformed) after allowing for the effects of sex and village (as before) using a regression analysis.

A description of these statistical methods has been published previously by Mutapi and Roddam [59]. All statistical tests were conducted using the software package SPSS and p values were taken to be significant at p<0.05.

## Results

### Schistosome epidemiology of study population

The prevalence of *S. haematobium* in the analysed population was 78% with a mean egg count of 28 eggs/10 ml urine (SEM = 5) with a range of 0 to 126 eggs/10 ml of urine. The population shows a typical convex age-infection profile for schistosome infection intensity, with infection intensity rising in the youngest age group (8–10 years), peaking in people aged 11–13 years and subsequently declining above 14 years of age. This infection profile corresponds to our data published previously for this population [47], [48].

**Figure 1:**
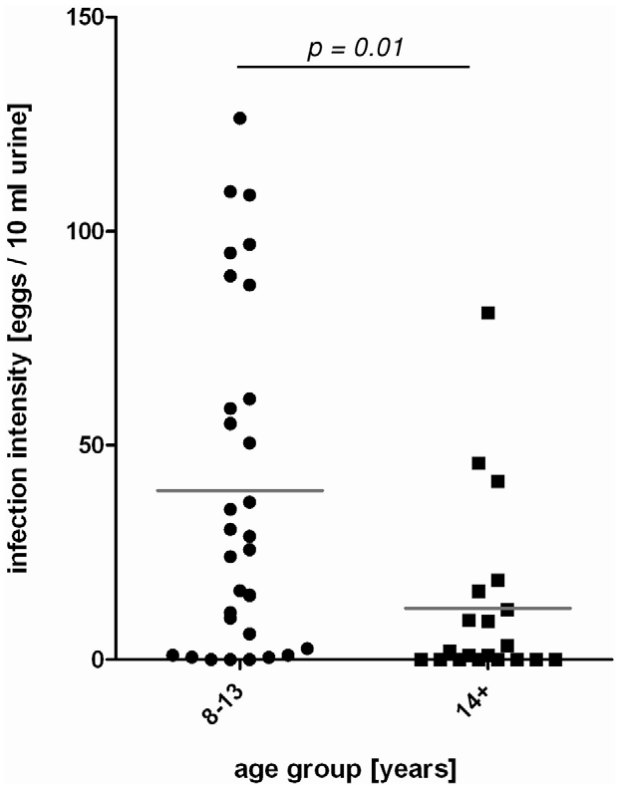
Study population was divided in two age groups. The 8–13 year age group (N = 30) infection intensity is rising and peaking, whereas in the 14+ year age group (N = 19) infection intensity is lower. Dots represent individual values of infection intensity to illustrate distribution within the age groups and gray line indicates means. Means were compared by Student's t-Test and p-value is shown.

Based on this profile the study population was divided into two age groups. In the first age group (8–13 years, N = 30) infection intensity is rising to peak whereas in the second group (14+, N = 19) infection intensity is declining as summarized in Figure 1. Mean infection intensity was significantly higher (p = 0.01) in the 8–13 year-group (39.4 eggs/10 ml urine ±7.3) compared to the 14+ year group (12.7 eggs/10 ml urine ± 4.9) and there were both egg negative and egg positive individuals in both groups as shown in Figure 1. The infection prevalence was significantly higher (χ^2^ = 5.16, df = 1, p = 0.023) in the younger group compared to the older group at 90% versus 63% respectively. All participants were negative for *Plasmodium* parasites as determined by microscopic examination of blood smears.

### Discrimination of regulatory and activated T cells

CD4^+^ Treg express a characteristic combination of molecular markers which distinguish them from activated effector T cells (Tact), including CD25^high^ and FOXP3 [38], [40]. In addition, Treg show diminished levels of CD4 [45] and low levels of CD127 [44].

**Figure 2:**
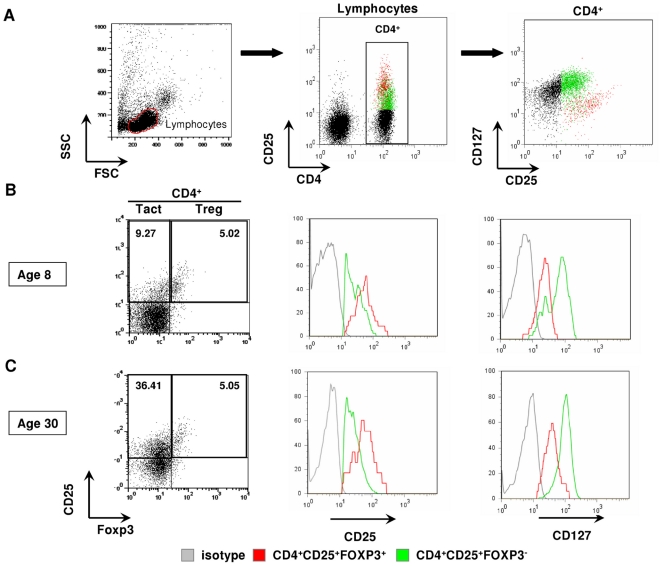
Treg and Tact are distinguished by a combination of several markers. (**A**) PBMC were electronically gated on lymphocyte population (left panel) followed by gate on total CD4^+^ cells (middle panel). CD4^+^ cells were analysed for the expression of CD25 and CD127 (right panel). (**A–C**) CD25^+^FOXP3^+^ cells were identified as Treg, whereas CD25^+^FOXP3^−^ counted as Tact. A dot plot for one individual is shown for group 1 (**B**) and group 2 (**C**). Gating on Treg and Tact subsets was verified by further analysis of level of expression of CD25 (**B–C**, middle panel) and CD127 (**B–C**, right panel) expressed on Treg (red) and Tact (green). Gray line - isotype control.

CD4^+^ lymphocytes were analyzed for the expression of CD25, FOXP3 and CD127 (Figure 2A) and gated on either CD4^+^CD25^+^FOXP^+^ (Treg) or CD4^+^CD25^+^FOXP^−^ (Tact) subsets (Figure 2B, C). CD25^+^FOXP3^+^ Treg expressed CD25 at high levels and showed a clear CD127^low^, CD4^dim^ phenotype, allowing the identification of Treg as CD4^+(dim)^CD25^+(high)^FOXP3^+^CD127^low^ cells (Figure 2A–C). In contrast Tact, identified as CD4^+^CD25^+^FOXP3^−^ cells, showed lower CD25 expression compared to the FOXP3^+^ cells. In addition most Tact expressed high levels of CD127 and normal levels of CD4. The expression of CD25^high^, CD4^low^, CD127^dim^ and FOXP3 were significantly correlated to each other (data not shown), and hence this combination of markers was used to discriminate between Tact and Treg populations.

The proportion of CD4^+^ T cells classified as Treg in this study ranged from 1.7 to 10.4% whereas, the proportion of CD4^+^ T cells classified as Tact ranged from 5.3 to 44.8%. A sample from each of the two age groups is shown in Figure 2B and C. These analyses revealed that the expression levels of CD25 and CD127 are comparable on Treg in different age groups whereas minor changes in the expression of both markers on Tact were observed in the youngest age group. Specifically, younger people have a small subset of FOXP3^−^ cells which show a CD127^dim^ CD25^high^ phenotype, these were not enumerated as Treg cells.

### The percentage of activated but not regulatory T cells changes with host age

**Figure 3:**
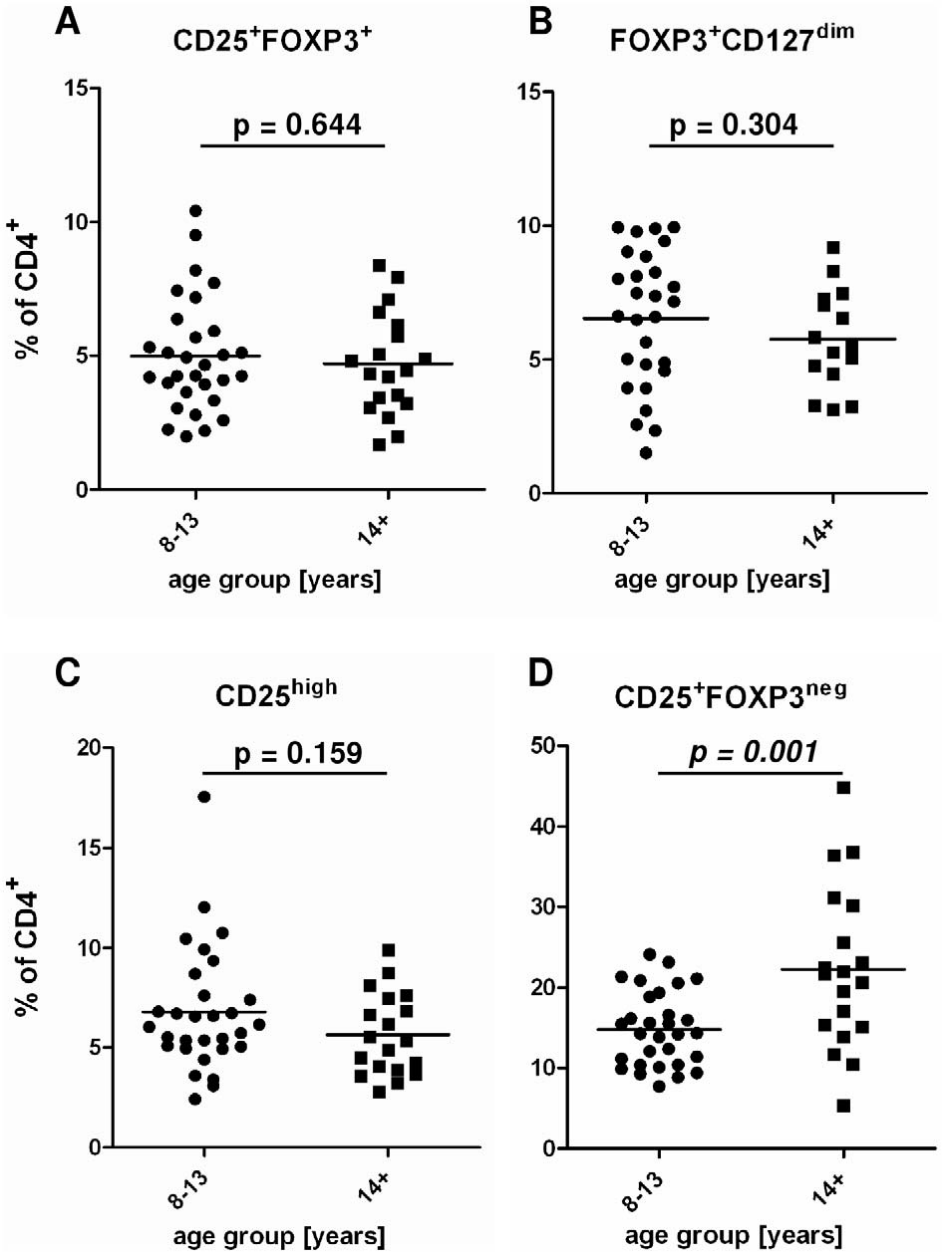
The proportion of Treg does not differ between age group regardless of markers employed. Dot plots show the fraction of Treg identified by a combination of different markers: (**A**) CD25^+^FOXP3^+^ cells, (**B**) FOXP3^+^CD127^dim^ cells and (**C**) CD25^high^ cells as percentage of CD4^+^ T cells in the different age groups. In (**D**) percentages of CD25^+^FOXP3^neg^ Tact are presented.

Since the T cell analysis focused on all T cells rather than schistosome-specific T cells, it was important to determine if the T cell populations varied with other host attributes. Sex did not affect proportions of CD4^+^CD25^+^FOXP3^+^ Treg and CD4^+^CD25^+^FOXP3^−^ Tact relative to CD4^+^ T cells (statistics presented in Table 1). The proportion of CD4^+^CD25^+^FOXP3^+^ Treg as percentage of total CD4^+^ T cell numbers did not change significantly between age groups as shown in Figure 3A. Expression of different markers of Tregs in the different age groups was also compared by analyzing for the proportion of CD4^+^ T cells which were FOXP3^+^CD127^dim^ cells (excluding CD25 as marker; Figure 3B) or the proportion of CD25^high^ cells (Figure 3C). Regardless of the marker combination used to identify Treg, no differences were observed between the two age groups (Figure 3A–C). However the percentage of activated T cells relative to total CD4^+^ T cells increased significantly with host age (Figure 3D and Table 1). The latter result meant that in subsequent analyses the confounding effects of age had to be allowed for before testing for the effects of schistosome infection intensity on the T cell populations.

**Table 1:** Influence of host attributes on T cell subsets.

Explanatory variable	Dependent variables	df	F-value	p-value
**sex**	Regulatory T cells	1, 47	0.345	0.560
	Activated T cells	1, 47	0.000	0.986
**age group**	Regulatory T cells	1, 47	0.105	0.748
	Activated T cells	1, 47	14.796	**<0.001**

Results from the multivariate analysis of variance determining the effect of host sex and age on percentages of activated (Tact) and regulatory (Treg) cells. df  =  degrees of freedom and tests significant at p<0.05 are highlighted in bold.

### The relationship between Treg and infection intensity varies with host age

**Figure 4:**
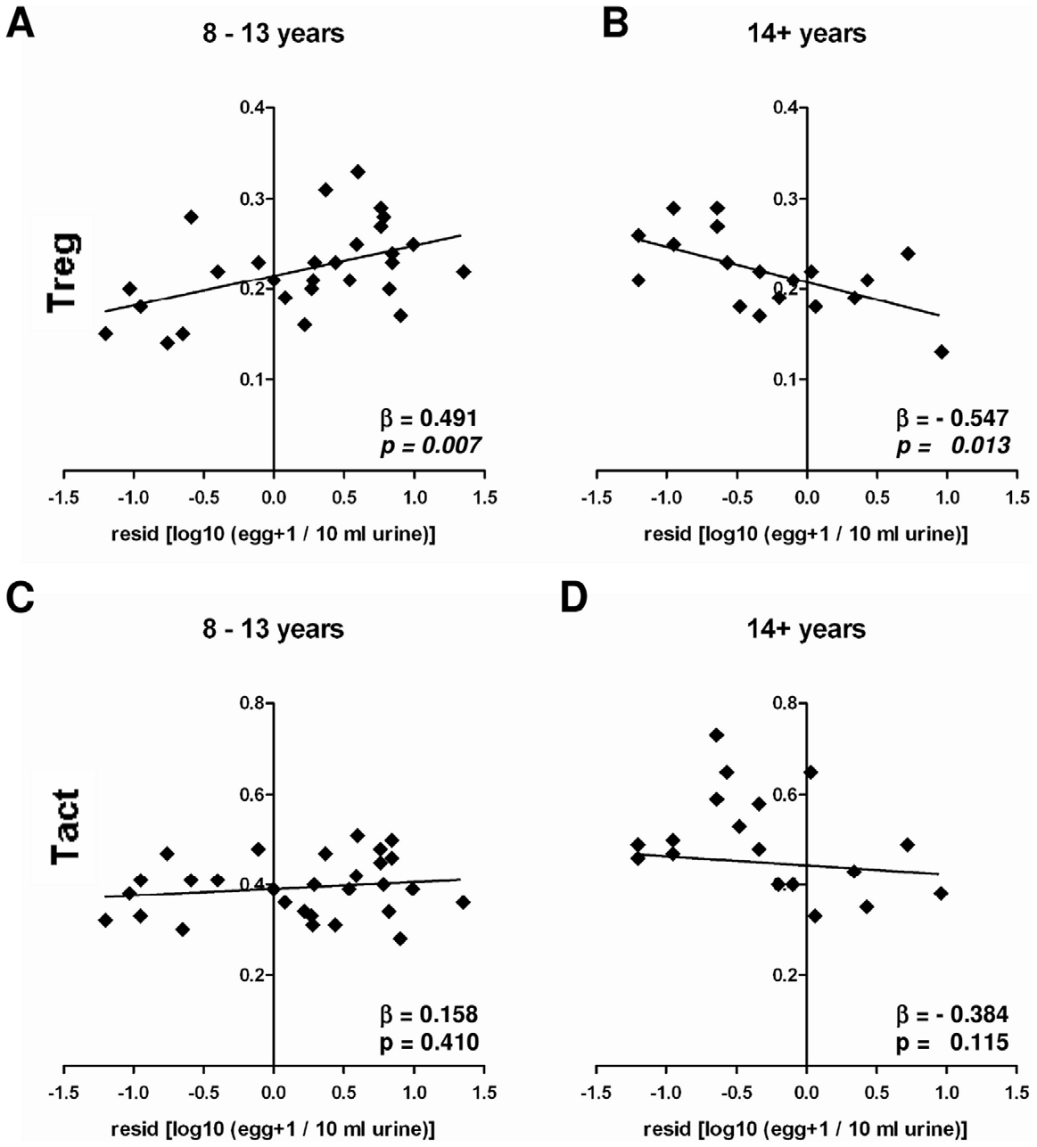
The association of Treg and Tact proportions with infection intensity are presented partitioned by age group. The variation in infection intensity ((log_10_ (egg count+1) transformed) was analysed for the potential confounding effects sex and village using ANOVA and calculated residuals are plotted against the proportion of Treg (**A, B**) or Tact (**C, D**; arcsin√ transformed) after dividing into two age groups as indicated. Obtained residuals were used for further used in a regression analysis whose p- and β- values are given.

The relationship between schistosome infection intensity and the percentage of regulatory and activated T cells to total CD4^+^ T cells was analyzed, after allowing for the effects of sex and host age in an analysis of variance. This analysis indicated that the relationship between Treg proportions and infection intensity varied between the two age groups (statistics presented in Table 2). In the young age group where infection was rising, the proportion of Treg of the total CD4^+^ T cell population increased significantly with increasing infection intensity (Figure 4A). The converse was true in the older age group where schistosome infection was declining, here the proportion of Treg declined with increasing infection intensity (Figure 4B). Grouping the participants by infection status (non-infected vs. infected as defined by the presence or absence of schistosome eggs in urine) rather than infection intensity produced similar results, so that in the younger age group infected participants had more Treg as proportion of CD4^+^ T cells than non-infected (p = 0.05), while in the older age group infected people had fewer Treg compared to non-infected (p = 0.02; data not shown). In contrast Tact showed no significant relationship with infection intensity (or infection status) in both age groups (Figure 4C, D).

**Table 2:** Relationship between infection intensity and Treg varies between age groups.

Dependent variables	Explanatory variable	df	F-value	p-value
Infection intensity	Tact	1, 47	0.638	0.429
	Treg	1, 47	2.983	0.092
	Tact * age group	1, 47	2.158	0.150
	Treg * age group	1, 47	10.031	**0.003**
	Treg * Tact	1, 47	0.185	0.669

Effects of sex and village of residence were allowed for and relation between infection intensity and T cells subsets was analysed independently and as function of age group. df  =  degrees of freedom and tests significant at p<0.05 are highlighted in bold.

## Discussion

An early paradigm of anti-helminth immune responses was based upon the Th1/Th2 dichotomy, with resistance to schistosome infection being associated with Th2 responses [5], [6]. Because it is now evident that the Th1/Th2 dichotomy does not sufficiently explain the balance between susceptibility and resistance to helminth infections (reviewed in [16]), there is increasing interest in the ability of regulatory T cells to modulate effector T cell responses. In mouse models, Treg play important roles in minimising pathology during schistosome infections [27], [34], and Treg depletion can restore immunity to filarial worm infections [15]. In human schistosomiasis, initial evidence for the association of regulatory T cells and infection was obtained by analysis of CD25^high^-expressing cell subsets [36], [60], showing a significant reduction in frequency of these cells following chemotherapeutic clearance of *S. mansoni* infection [36]. We conducted our studies on total populations of Treg and Tact cells rather than parasite-specific T cells to determine if an association with schistosome infection could be determined at the T cell population level. This is in keeping with the paradigm of helminth infections stimulating regulatory responses which affect responses to unrelated antigens by affecting effector T cell responses [61]. Therefore our objective was to determine the association of schistosome infection and these two T cell populations.

In this study we were able to characterise naturally occurring regulatory T cells in a human helminth infection with a more comprehensive marker set, namely CD4^+(dim)^CD25^+(high)^FOXP3^+^CD127^low^ cells and analysed the relationship to activated T cells designated as CD4^+^CD25^+^FOXP3^−^. First we observed that the proportions of Treg relative to CD4^+^ T cells in this population are slightly higher than those reported in populations not exposed to schistosomiasis as reported elsewhere [35], [45], but comparable to those reported for Kenyans exposed to *S. mansoni* [36] suggesting a relationship between exposure to the helminth infection and the development of Treg cells. Percentages of Tact were significantly higher in the older age group compared to the younger individuals. This is in accordance with data showing that CD4^+^ T cells with effector/memory, which encompass activated T cells, increase with age, a change which can be already observed below the age of 20 [62]. In contrast proportions of Tregs relative to CD4^+^ T cells did not differ significantly between the two age groups.

The most striking finding of this study was that the correlation between Treg proportion and infection intensity of *S. haematobium* differed significantly between the two age groups. After controlling for the confounding factors of age (and thus age related biological processes) we demonstrated that Treg as percentages of total CD4^+^ T cells were correlated with infection intensity, being significantly positive in the younger age group where infection was rising and peaking. In contrast the converse was true in the older age group where higher infection intensity was associated with lower Treg numbers. However there was no significant association between the infection intensity and Tact. The consequence is an increase of the ratio of regulatory to activated T cells with infection intensity in younger people (dictated by Treg) which may suggest a down-regulation of effector responses, but also may protect against immunopathology [27], [33], [63]. In contrast the correlation between proportions of regulatory T cells and level of infection turned negative in the older age group in whom infection levels were declining, suggesting that older individual carrying infection might be more prone to developing the characteristic immunopathology of chronic schistosomiasis.

However the relationship between different levels of Treg relative to infection intensity and the development immunopathology remains to be established. Furthermore detailed studies of parasite-specific T cell and functional characterization will clarify the nature and development of responses associated with resistance to human schistosome infections.
